# Seroprevalence and Risk Factors for Equine West Nile Virus Infections in Eastern Germany, 2020

**DOI:** 10.3390/v14061191

**Published:** 2022-05-30

**Authors:** Stefanie Ganzenberg, Michael Sieg, Ute Ziegler, Martin Pfeffer, Thomas W. Vahlenkamp, Uwe Hörügel, Martin H. Groschup, Katharina L. Lohmann

**Affiliations:** 1 Department for Horses, Faculty of Veterinary Medicine, Leipzig University, 04103 Leipzig, Germany; stefanie.ganzenberg@vetmed.uni-leipzig.de; 2 Institute of Virology, Faculty of Veterinary Medicine, Leipzig University, 04103 Leipzig, Germany; michael.sieg@vetmed.uni-leipzig.de (M.S.); vahlenkamp@vetmed.uni-leipzig.de (T.W.V.); 3 Friedrich-Loeffler Institut (FLI), Federal Research Institute for Animal Health, Institute of Novel and Emerging Infectious Diseases, 17493 Greifswald-Insel Riems, Germany; ute.ziegler@fli.de (U.Z.); martin.groschup@fli.de (M.H.G.); 4 Institute of Animal Hygiene and Veterinary Public Health, Faculty of Veterinary Medicine, Leipzig University, 04103 Leipzig, Germany; pfeffer@vetmed.uni-leipzig.de; 5 Animal Diseases Fund Saxony, Pferdegesundheitsdienst, 01099 Dresden, Germany; uwe.hoeruegel@tsk-sachsen.de

**Keywords:** West Nile virus, horses, seroprevalence, Germany, epidemiology, risk factors, tick-borne encephalitis virus, Usutu virus

## Abstract

West Nile virus (WNV) infections were first detected in Germany in 2018, but information about WNV seroprevalence in horses is limited. The study’s overall goal was to gather information that would help veterinarians, horse owners, and veterinary-, and public health- authorities understand the spread of WNV in Germany and direct protective measures. For this purpose, WNV seroprevalence was determined in counties with and without previously registered WNV infections in horses, and risk factors for seropositivity were estimated. The cohort consisted of privately owned horses from nine counties in Eastern Germany. A total of 940 serum samples was tested by competitive panflavivirus ELISA (cELISA), and reactive samples were further tested by WNV IgM capture ELISA and confirmed by virus neutralization test (VNT). Information about potential risk factors was recorded by questionnaire and analyzed by logistic regression. A total of 106 serum samples showed antibodies against flaviviruses by cELISA, of which six tested positive for WNV IgM. The VNT verified a WNV infection for 54 samples (50.9%), while 35 sera neutralized tick-borne encephalitis virus (33.0%), and eight sera neutralized Usutu virus (7.5%). Hence, seroprevalence for WNV infection was 5.8% on average and was significantly higher in counties with previously registered infections (*p* = 0.005). The risk factor analysis showed breed type (pony), housing in counties with previously registered infections, housing type (24 h turn-out), and presence of outdoor shelter as the main significant risk factors for seropositivity. In conclusion, we estimated the extent of WNV infection in the resident horse population in Eastern Germany and showed that seroprevalence was higher in counties with previously registered equine WNV infections.

## 1. Introduction

First isolated from the blood of a febrile woman in the West Nile District of Uganda in 1937 [1], the West Nile virus (WNV) is today considered one of the most widely distributed flaviviruses worldwide [2]. It has caused human and animal disease outbreaks in all continents, except Antarctica [3].

WNV is an arthropod-borne, single-stranded, positive-sense RNA virus, and serologically belongs to the Japanese encephalitis virus serocomplex within the *Flaviviridae* family [4]. Next to WNV, the complex includes the Usutu virus (USUV), Japanese encephalitis virus (JEV), St. Louis encephalitis virus (SLEV), and Murray Valley encephalitis virus (MVEV) [5]. Recent phylogenetic analyses revealed a subdivision of WNV into up to nine lineages, of which lineages 1 and 2 are the most recognized [6] Lineage 1 isolates have a worldwide distribution and caused significant outbreaks, including the 1999 epidemic in the USA [7]. Lineage 2 strains initially were associated with asymptomatic infections. Since first being isolated from birds in Hungary in 2004 and 2005 [8], lineage 2 WNVs gained substantial epidemic potential and caused numerous outbreaks in Europe [9].

WNV is maintained by an enzootic cycle between susceptible bird species and competent mosquitoes, particularly *Culex* species mosquitoes [9], as vectors. A wide variety of mammals and reptiles [10,11,12], including humans and horses, can occasionally become infected with WNV. Generally, these species do not develop sufficient viremia to sustain transmission and are considered incidental or dead-end-hosts [13,14].

While most WNV infections in horses are subclinical, up to 8% can lead to clinical disease [13,15,16]. Clinical signs resulting from natural or experimental infections in horses range from lethargy and fever to various neurologic symptoms including ataxia, muscular weakness of the limbs (paresis or paralysis), recumbency, muscle fasciculations, altered mental state, hyperesthesia and cranial nerve abnormalities [17,18,19,20,21,22]. In reported outbreaks, mortality rates in infected horses with clinical signs ranged from 23% to 43% [23,24,25], with approximately 80% of clinically affected survivors fully recovering [26]. No specific drug or therapy is currently licensed to treat WNV infections [27,28]. While there is no approved vaccine for humans to date [29,30], three vaccines are approved in Europe for the vaccination of horses. The licensed vaccines contain a West Nile recombinant canarypox virus [31], a chimeric yellow fever-West Nile vector [32], or inactivated West Nile virus [33], respectively. Efficacy of the vaccines has been proven in field and laboratory studies, mainly by demonstrating reduced viremia or reduced duration or intensity of clinical signs, in vaccinated horses [16,34,35,36,37,38,39]. The German Commission on Vaccination in Veterinary Medicine (Ständige Impfkommission Veterinärmedizin) recommends WNV vaccination for horses kept in or moved to known risk areas [40]. Immunization should be completed before the beginning of the mosquito season in early May.

A first considerable increase in reported outbreaks in Europe occurred with the introduction of lineage 2 isolates in the late 2000s [41]. In addition to outbreaks in southern Europe [42,43,44,45,46,47], equine WNV infections had been confirmed in countries neighboring Germany such as Austria [48], the Czech Republic [49,50], and Poland [51,52]. In 2018, the number of registered WNV infections in Europe increased significantly compared to previous years, with 2083 human and 285 reported equine cases [53]. Simultaneously WNV was detected for the first time in captured birds and two horses in Germany [54]. For 2019, 36 WNV infections were registered in horses and numbers remained high in 2020 and 2021, with 22 and 19 reported cases, respectively [55]. Except for one, all WNV infections in horses were registered in Eastern central Germany in Berlin and the federal states of Brandenburg, Saxony-Anhalt, Saxony, and Thuringia.

In Germany, WNV infections in birds and horses have been classified as notifiable animal disease since 2009 [56]. In the absence of a national statutory case definition, the reference laboratory for WNV at the Friedrich-Loeffler-Institute refers in its definition of a reportable case [57] to the definition of the World Organization for Animal Health —OIE [58]. The geographical distribution of registered equine infections in Germany since 2018 suggests circulation of WNV in Eastern Germany; however, information on virus spread within the German horse population is limited. One very early study from 2007 to 2009 combined a limited number of horse samples with the investigation of sera from wild birds and free-ranging poultry in selected regions of the country [59]. The second study, from 2010 to 2012, focused primarily on equine infectious anemia in Germany, but also examined more than 5000 sera for possible WNV infection [60]. Neither study reported WNV-specific antibodies in the investigated horses, although serological cross-reactions with tick-borne encephalitis virus (TBEV) were described. A recent serosurvey study after the introduction of WNV into Germany showed the establishment of WNV infections in the horse population in Berlin/Brandenburg with a seroprevalence rate of 8.16% in 2019 and 13.77% in 2020, while no WNV circulation could be demonstrated in the Western part of the country [61].

The cross-sectional study presented here aimed to estimate the seroprevalence of equine WNV infections in an area with known WNV circulation and to investigate regional differences among counties with and without registered equine WNV infections. In addition, potential risk factors for infection at the animal, housing, and management levels were investigated.

## 2. Materials and Methods

### 2.1. Ethical Statement

Ethical approval for the study was obtained from the ‘Landesdirektion Sachsen (Saxony)’ (Nr. A06/20), the ‘Landesamt für Arbeitsschutz, Verbraucherschutz und Gesundheit Brandenburg’ (Nr. 2347-A-33-1-2020), and the ‘Landesverwaltungsamt, Referat Verbraucherschutz, Veterinärangelegenheiten Sachsen-Anhalt (Saxony-Anhalt)’ (AZ: 42502-3-892KlinikPferd). Horse owners were required to sign an informed consent form prior to participating in the study.

### 2.2. Study Area and Animals

**Figure 1:**
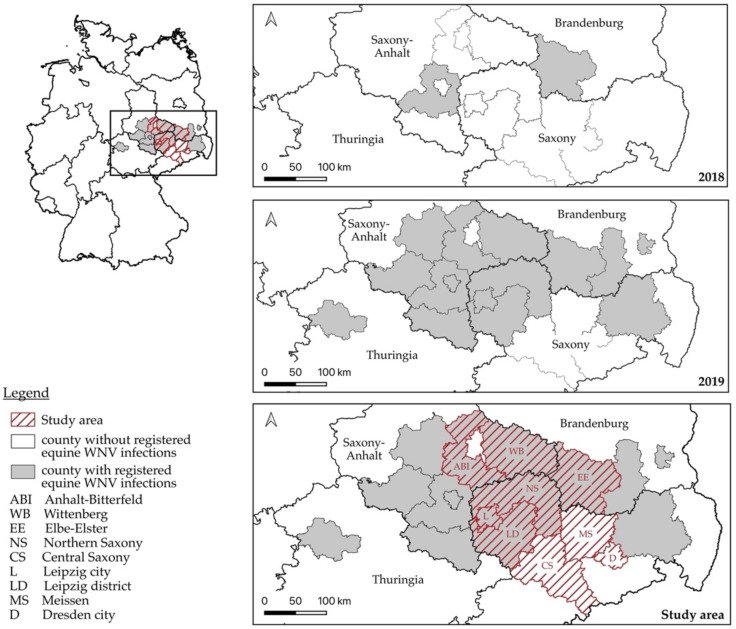
Counties in Eastern Germany with registered equine WNV infections (shaded in grey) in 2018 (**top**), and 2019 (**middle**), and the selected study area for estimating WNV seroprevalence in 2020 (**bottom**). In the bottom map, counties with registered WNV infections in previous years are shaded in grey, while the study area is hatched in red. The study area comprised six counties in Saxony (NS, L, LD, CS, MS, D), two counties in Saxony-Anhalt (ABI, WB), and one county in Brandenburg (EE), and included six counties with and three counties without previously registered equine WNV infections.

The survey area in Central Eastern Germany was selected based on the registered equine WNV infections in 2018 and 2019. It extended over 13,156 km^2^ and included nine counties in three federal states: Anhalt-Bitterfeld (ABI) and Wittenberg (WB) in Saxony-Anhalt, Elbe-Elster (EE) in Brandenburg, and Northern Saxony (NS), Central Saxony (CS), Leipzig city (L), Leipzig district (LD), Meissen (MS) and Dresden city (D) in Saxony. Six counties (ABI, WB, EE, NS, L, LD) had registered equine WNV infections in previous years, while three (CS, MS, D) had not (Figure 1). Geographically, the study area was located at the southern end of the North German plain (52°10′ N–50°60′ N and 11°80′ E–13°90′ E) with the main altitude between 50 and 450 m above sea level. With a measured annual average temperature of 10.4–11.1 °C and an average precipitation rate between 485.4 and 602.3 mm^3^ per year [62], the region represented a moderate continental climate.

Based on a population of approximately 22,000 registered horses in the study area as of June 2020, an expected seroprevalence of approximately 10%, a confidence interval of 95%, and a margin of error of 2%, a required sample size of at least 832 horses was calculated using Epi Info^TM^ [63]. Participants were recruited from equine holdings registered according to Regulation (EU) 2016/429, Article 12. Owners (Holdings) with at least five registered horses were invited to participate in the study. Horses (including donkeys and mules) enrolled in the study had to be at least 12 months of age, unvaccinated against the WNV, and permanently kept in the area. Age and vaccination status were verified for all enrolled horses by reviewing their passports.

### 2.3. Sample Processing

Blood samples were taken by jugular vein puncture using a sterile vacuum collection system (Vacuette^®^, Greiner Bio-One GmbH, Frickenhausen, Germany) and an 18-gauge needle. Samples were labeled, transported chilled to the laboratory, and refrigerated at 4 °C until further processing. Within 24 h of collection, samples were centrifuged at 400× *g* at 10 °C for 10 min, and serum was aliquoted and stored at −20 °C until further analysis.

### 2.4. Epidemiological Data

For each horse enrolled in the study, data were collected from the holding’s manager or horse owner by filling out a standardized questionnaire through an on-site interview (Figure S1 in the Supplemental Data). Recorded variables included: (i) At the animal level: age (in years); breed type (draft horse, Warmblood, Thoroughbred, pony, donkey and mule); sex (mare, stallion, gelding); coat color (dark [black, dark bay, bay], chestnut, light [roan, dun, palomino], very light or white [Cremello, white], spotted [Tobiano, Overo, Appaloosa]); country of birth; primary use (leisure, sport horse, breeding, retired); primary training location (exclusively in the arena, mainly in the arena, mainly trail riding, exclusively trail riding, no training); travel outside of Germany in the previous two years (yes, no); clinical signs of neurologic disease in the previous two years (yes, no); transport at a distance of more than 20 km from the home stable within the last year (yes, no). (ii) At the housing level: location of the holding (address); number of horses within the holding; type of housing during the mosquito season (stabled without turn-out, stabled with turn-out time <12 h/day, stabled with turn-out time >12 h/day, permanent outdoor housing); type of turn-out (dry lot, pasture, combination of both); number of additional horses within the turn-out; presence of outdoor shelter (yes, no); presence of stagnant water within one km of the holding (yes, no); percentage of WNV-vaccinated horses within the holding. (iii) Mosquito control measures: estimated number of mosquitoes (massive numbers, a lot, few, none); use of insect repellent (yes, no) and, if yes, frequency of application (always, mostly, rarely, never) and time of application (while stabled, while working, in the turn-out, during transport); type of insect repellent (homemade, commercial, both) as well as active ingredient or brand; use of a flysheet (yes, no) and, if yes, type of flysheet (regular, zebra print, sweet itch sheet); type of water supply (automatic waterers, buckets or troughs), weekly water change in troughs (yes, no); additional mosquito control measures (PVC strip curtains, fly screens in windows, horsefly traps, electric insect traps, traps with attractant, sticky tape fly traps).

### 2.5. Serology

Serum samples were initially tested by a panflavivirus competitive enzyme-linked immunosorbent assay (cELISA) (ID Screen^®^ West Nile Competition Multi-species; IDvet Innovative Diagnostics, Grabels, France). All analyses were run in duplicate, according to the manufacturer’s instructions. To detect acute infections, cELISA-positive and -equivocal samples were further tested by an IgM capture ELISA (ID Screen^®^ West Nile IgM Capture; IDvet Innovative Diagnostics, France).

To rule out cross-reactivity with antibodies against relevant flaviviruses (Usutu virus, USUV, and tick-borne encephalitis virus, TBEV), all cELISA-positive or -equivocal samples were re-tested by micro-virus neutralization test (VNT) in duplicate, using a previously published protocol [64]. Briefly, dilutions of heat-inactivated sera were incubated at 37 °C for one hour with equal volumes of 100 TCID_50_ of a WNV-lineage 2 strain (WNV strain Germany, Gen-Bank accession no. MH924836), TBEV strain Neudoerfl (kindly provided by G. Dobler, Bundeswehr Institute of Microbiology, Munich, Germany; GenBank accession no. U27495) or USUV strain Germany (Europa 3, GenBank accession no. HE599647) before addition to wells containing monolayers of target cells. Seven days after infection, observable cytopathic effects were recorded. As calculated by the Behrens–Kaerber method, the neutralizing titer (ND_50_) was defined as the reciprocal of the maximum dilution that inhibited cytopathic effects in 50% of the wells, and neutralizing titers of 10 or higher were considered positive. For the purpose of further analysis, serum samples were considered seropositive for antibodies against WNV, USUV, or TBEV based on the results of the VNT. When neutralizing effects against more than one virus were detected, the serum was deemed positive for antibodies against the virus that was neutralized at a four-fold higher dilution than the other viruses. If a four-fold difference in neutralizing titers was not evident, the results were considered inconclusive, and the sample was excluded from further analysis. Samples testing positive by cELISA but negative by VNT were considered seronegative.

### 2.6. Statistical Analysis

Seroprevalence of WNV infection at the animal level was calculated as the number of WNV-seropositive animals among the tested horses, and seroprevalence at the holdings level was calculated as the number of holdings with at least one seropositive horse among the total number of holdings.

To evaluate risk factors for seropositivity, univariate analysis using chi-square statistics were initially carried out for all variables. In a second step, a logistic regression model was performed to predict the log-likelihood of the outcome, positive WNV–VNT, as an additive function of potential risk factors (variables). To avoid bias in the analysis due to the exclusion of individual subgroups, only variables with valid values in all categories were considered. For some variables, categories specified in the questionnaire were, therefore, re-categorized as follows: (i) for country of birth: Germany, WNV-endemic areas until 2019 (Poland, Czech Republic, Hungary, Austria, Spain, France, USA) or WNV- free areas until 2019; (ii) for the type of housing during mosquito season: housing with 24 h access to turn-out (permanent outdoor housing) or housing with less than 24 h access to turn-out; (iii) for counties: counties with registered equine WNV infections in 2018 and 2019, or counties with no registered equine WNV infections in 2018 and 2019. Subordinate variables, which overlapped within their categories due to the option of multiple answers, were not included in the regression model. Twenty-two remaining predictor variables were entered into the final model and subjected to dummy coding, if non-dichotomous. All metric variables were entered into the model as continuous variables. The measure of effect strength was given in Odds. Odds ratios (OR) with *p* < 0.05 were considered significant.

The measure of association between significant variables was calculated using Cramér’s V or Pearson correlation coefficient. Cramér’s V values range from 0 to 1, where 0 indicates no association between the two variables and 1 corresponds to one variable being entirely determined by the other. According to Cohen’s interpretation of Cramér’s V [65], values of V < 0.1 are defined as a negligible association, values between 0.1 and 0.3 as weak, values between 0.3 and 0.5 as moderate, and values of 0.5 and higher as a strong association. Since nominal data were used for the calculations, results pertain only to the strength but not the direction of the relationship. All statistical analyses were performed using IBM SPSS Statistics version 27.0 (SPSS Inc., Chicago, IL, USA).

### 2.7. Mapping

Maps were created using QGIS (QGIS Geographic Information System, Odense 3.20, Gary E. Sherman et al., Boston, MA, USA). Geographical locations were based on Google-derived GPS coordinates of the holdings (Google Maps, 2020, maps.google.de).

## 3. Results

### 3.1. Population and Questionnaire

All sampling took place at the end of the WNV-transmission season between September and November 2020. Of 21,882 horses registered on 7713 holdings in the survey area in 2020, 10,190 horses were kept on 928 holdings with at least five horses and met the inclusion criteria. Out of those, 940 horses (4.3% of all registered horses; 931 horses, nine donkeys) from 127 holdings were enrolled. Sixty-point three percent of the enrolled horses originated from Saxony, 25.2% from Saxony-Anhalt, and 14.5% from Brandenburg. The included population by county ranged from 2.4% in Leipzig city to 7.2% in Wittenberg and Dresden city (Table 1). Approximately two-thirds of the samples (*n* = 641, 68.2%) were taken in counties with registered equine WNV infections in previous years.

Most sampled horses were Warmbloods (*n* = 554, 59.1%,), followed by ponies (*n* = 287, 30.6%), Thoroughbreds (*n* = 49, 5.2%), draft horses (*n* = 38, 4%) and donkeys (*n* = 9, 1%). The sex distribution showed slightly more females (*n* = 536, 57.2%), than males (*n* = 62 stallions and *n* = 339 geldings, 42.8%). Age ranged from 1–33 years with a mean of 12.8 ± 7.8 years. Ninety-two percent (*n* = 862) of the sampled horses were born in Germany, 3.7% (*n* = 35) originated from WNV-endemic countries, and 3.8% (*n* = 36) from countries with no evidence of WNV infections. For 0.4% (*n* = 4) of the horses, the country of birth was unknown. Leisure horses represented the largest subgroup (*n* = 390, 41.6%) of the cohort, while 30.3% (*n* = 284) were described as sport horses, and 19.4% (*n* = 182) were primarily used for breeding. Sixty-six-point six percent (*n* = 624) of all horses were permanently housed outside during mosquito season (April to November), while 22% (*n* = 206) were stabled with a turn-out time of fewer than 12 h/day, and 11.4% (*n* = 107) were stabled with more than 12 h/day of turn-out.

Holdings housed between two and 160 horses (mean: 15.5, median: nine), and more than 75% of the holdings (*n* = 97) housed less than 20 horses. In each holding, between one and 39 horses were sampled (mean: 7.4, median: 6), representing between 1.3% and 100% of the horses on the holding (mean: 67.7%; median: 75%). The turn-out was described as pasture for 63.4% (*n* = 594) of the equines, dry lot for 10.4% (*n* = 97), and both for 26.3% (*n* = 246). A shelter in the turn-out existed for less than half of the sampled horses (*n* = 436, 46.5%). Between 0% and 89.5% of horses on the enrolled holdings were vaccinated against WNV (mean: 15%, median: 0%), with 58.4% of the enrolled horses (*n* = 547) kept on holdings with 0% vaccination rate, 31.7% (*n* = 297) on holdings with less than 50% vaccination rate, and 9.9% (*n* = 93) on holdings with more than 50% vaccination rate against WNV.

Insect repellents were used to protect against mosquitoes in 56.9% (*n* = 533) of the enrolled horses, with most owners (*n* = 472, 50.4%) reporting at least occasional use while working their horses. Repellents were used in 29.4% (*n* = 275) of the horses during turn-out and in 6.8% (*n* = 64) while stabled. Use of fly sheets was only reported for 99 horses (10.6%), and types included 50 regular blankets (5.3%), 31 blankets with a zebra pattern (3.3%), and 18 sweet itch sheets (1.9%). Slightly more than a quarter of the horses (*n* = 250, 26.7%) were kept on holdings with additional mosquito control measures (PVC strip curtains, fly screens in windows, or fly traps). The complete data of the enrolled holdings and descriptive statistics are shown in Tables S1 and S2 of the Supplemental Data.

### 3.2. Serology

As analyzed by cELISA, 102 of 940 (10.9%) sera tested positive for flavivirus-specific antibodies, with six of those samples also testing positive for WNV IgM antibodies (0.6%). Four samples were considered equivocal, such that 106 (11.3%) samples were submitted for VNT. Based on the VNT, 54 sera (5.8%) were positive for WNV-neutralizing antibodies (ND_50_ titers ranged from 10 to 1920), 35 sera (3.7%) showed specific antibodies against TBEV (ND_50_ titers ranged from 40 to 2560), and eight sera (0.9%) showed specific antibodies against USUV (ND_50_ titers ranged from 10 to 160). Two samples showed neutralizing antibodies against TBEV and WNV, and one sample reacted positively for neutralizing antibodies against USUV and WNV, without a four-fold difference in neutralizing titers. Consequently, these three samples were categorized as inconclusive and excluded from further analysis. Despite positive cELISA results, no neutralizing antibodies against any of the tested viruses were detected in six samples, which were, therefore, considered seronegative. Complete results of the serological analysis are presented in Table S3 in the Supplemental Data.

### 3.3. Seroprevalence

**Figure 2:**
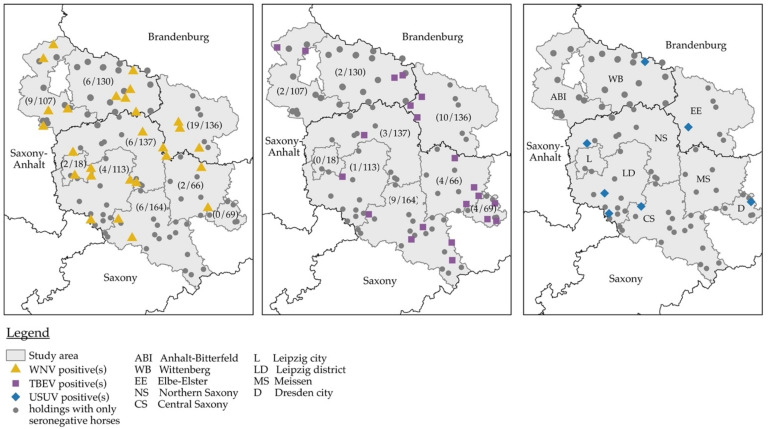
Distribution of seropositive and seronegative horses within the study area. Symbols indicate holdings that can house one or more seropositive horse(s). The numbers in brackets indicate the number of seropositive horses for WNV and TBEV per total number of samples in the respective county. For USUV, each symbol indicates one seropositive horse sample, except for Dresden, where two seropositive horses originated from one holding.

The complete seroprevalence data for WNV infection can be viewed in Table 1 top (on the horse level) and Table 1 bottom (on the holdings level). The distribution of seropositive and seronegative horses within the study area is depicted in Figure 2.

Based on the VNT results, the overall seroprevalence for WNV infection in unvaccinated horses was 5.8% (54/937) and ranged from 0% (0/69) in Dresden to 14.0% in Elbe-Elster (19/136). None of the WNV-seropositive horses had stayed outside of Germany during the two years prior to sampling. Two horses were born in countries with known WNV activity in the horse population (Czech Republic, Poland); the horse born in Poland had been imported to Germany more than two years before sampling, while information concerning the time of import was not available for the second horse. All the other WNV-seropositive horses (*n* = 48) were bred in Germany or in countries without registered WNV outbreaks among humans or animals (UK, *n* = 1; the Netherlands, *n* = 2) or without evidence of an endemic WNV-situation before 2020 (Switzerland, *n* = 1), according to the European Centre for Disease Prevention and Control, ECDC [66]. Seropositive horses were detected in eight of the nine counties surveyed, and a significant association existed between county and WNV seropositivity (χ^2^ = 27.4; df = 8; *p* = 0.001). Seroprevalence in counties with registered equine WNV infections in 2018/2019 was significantly higher, than in those without registered infections (7.2% vs. 2.7%; χ^2^ = 7.7, df = 1, *p* = 0.005).

Twenty-seven of the 127 holdings included in the study (21.3%) housed at least one WNV-seropositive horse. The size of holdings with seropositive horses varied between four and 80 horses (mean: 19.8, median: 11), and between 0% and 100% (7/7) of the tested horses on a holding were seropositive. Seroprevalence at the holdings level was similarly distributed as seroprevalence at the animal level, with the highest values detected in Elbe-Elster, Leipzig city, and Anhalt-Bitterfeld, and the lowest values detected in Central Saxony and the city of Dresden.

### 3.4. Clinical Signs

Of the six horses testing positive for IgM antibodies against WNV, clinical signs of neurologic disease were reported for one horse (ataxia, stiff gait, involuntary muscle fasciculations) approximately four to six weeks prior to sampling. According to the owner, the horse received no treatment and had no clinical signs while participating in the study. The other five horses that had tested positive for WNV IgM antibodies had not shown any neurological signs in the two years prior to sampling. Except for two horses with registered WNV infection in 2019, none of the remaining horses that tested positive by cELISA had a history of showing neurologic signs consistent with WNV infection.

### 3.5. Risk Factor Analysis

Significant variables of the univariate analysis are shown in Table 2 and complete results are available in Table S4 of the Supplemental Data.

The binomial logistic regression model for the outcome of WNV seropositivity was statistically significant (χ^2^ = 88.4, df = 37; *p* < 0.001), resulting in an acceptable level of explained variance [67], as shown by Nagelkerke’s R^2^ = 0.253. The overall percentage of accuracy in classification was 72.1%, with a sensitivity of 72.1% and a specificity of 72.2%. Of the 22 explanatory variables entered in the regression model, six significantly predicted seropositivity (Table 3). Breed type pony (OR = 0.29; *p* = 0.013) and increasing WNV vaccination density in the operation (OR = 0.97; *p* = 0.01) reduced the likelihood of seropositivity, while being located in a county with previously registered equine WNV infections (OR = 3.91; *p* = 0.003), permanent outdoor housing (OR = 2.63; *p* = 0.033), the existence of a shelter in the turn-out (OR = 3.02; *p* = 0.01) and the usage of a fly sheet (OR = 7.22; *p* < 0.001) increased the likelihood of seropositivity. Complete results are available in Table S5 of the Supplemental Data.

Weak associations between breed type and the county where horses were registered, and between breed type and the type of housing, were detected. Fewer ponies than predicted lived in counties with higher WNV seroprevalence (Cramer-V = 0.24, *p* < 0.001), while more ponies than statistically predicted were permanently kept outside (Cramer-V = 0.14, *p* = 0.001). Type of housing and type of turn-out showed a negligible association (Cramer-V = 0.081, *p* = 0.045). A statistically moderate association between the type of housing and the existence of a shelter in the turn-out was present (Cramer-V = 0.381, *p* < 0.001). Horses permanently housed outside had a shelter more often than expected, while those with less than 24 h access to turn-out had a shelter less often than statistically expected. Vaccination density showed a moderate positive correlation with the number of horses kept on the holding (Pearson correlation = 0.3, *p* < 0.001). Fly sheet use was associated with the estimated number of mosquitoes present. Owners reporting a lot of, or massive numbers of, mosquitoes on the holding used a fly sheet more often than statistically expected (Cramer-V = 0.271, *p* < 0.001). There was no association between the use of a fly sheet and the type of housing. Horses kept outside permanently were more frequently treated with self-made repellent if a repellent was used (Cramer-V = 0.079, *p* < 0.015). No correlation existed between the housing type and the usage of a commercial spray.

## 4. Discussion

In addition to estimating an overall seroprevalence of 5.8% at the horse level and 21.3% at the holdings level, we were able to show significant differences between counties as well as a higher seroprevalence in counties with registered equine WNV infections in previous years (2018/2019). The estimated WNV seroprevalences are comparable to those reported for several European countries in the last decade. For instance, a seroprevalence of 6.4% at the horse level and 17.8% at the herd level was reported in Italy [43], while seroprevalence was 7.1% at the horse level and 8.3% at the herd level in Andalucía, Spain [68]. Comparable seroprevalence on horse level of 4.1% in the Czech Republic [50], 5.3% in Austria [69] and 3.4% in Croatia [47] have been reported. Higher seroprevalences were observed in Poland (15.1%) [52] and Portugal (15.0%) [46], while seroprevalence of WNV infections in the United Kingdom was 0% in one study in 2019 [70]. To our knowledge, WNV infections in horses have not been reported for other countries bordering Germany, including Switzerland, the Scandinavian countries, or the Netherlands, although infections in humans, birds, and mosquitoes have been described [71,72]. Our study also supplements the results of Bergmann et al. [61], who reported seroprevalence of equine WNV infections in a hospitalized population in Berlin/Brandenburg and North Rhine-Westphalia. The WNV seroprevalence for 2020 in horses from Berlin/Brandenburg determined in that study (13.77%) corresponds approximately to the WNV seroprevalence (14.0%) for the district of Elbe-Elster (Brandenburg) determined in the present survey.

The cohort evaluated here represented 4.8% of registered horses and 9.2% of eligible horses in the study area, respectively, since, for practical reasons, we only invited holdings with at least five registered horses to participate in the study. Within the examined cohort, females were slightly overrepresented, the age range was broad and, subjectively, the most common breed types and coat colors were represented. Sampled horses and holdings were relatively evenly distributed throughout the study area (Figure 2). Therefore, our results present a reliable estimate of WNV seroprevalence, although additional studies are needed to investigate the seroprevalence in other parts of the country and to judge any further spread of WNV in Germany. The mean vaccination density of 15% on the investigated holdings has to be interpreted with caution as we included only horses not vaccinated against the WNV in the study. This number may underestimate actual vaccination coverage, e.g., if horse owners with a large proportion of vaccinated horses chose not to participate in the study. Conversely, the number may be an overestimate if one assumes that horse owners interested in participating in research are more invested in the health of their horses and more likely to vaccinate. Keeping these limitations in mind, we consider the determined vaccination density found here to be rather high, given the fact that WNV infections in horses have only recently become a problem in Germany.

The detection of neutralizing antibodies against WNV in resident horses in eight out of nine surveyed counties supports virus circulation in these regions. The fact that none of the WNV-positive horses traveled outside of Germany in the 24 months prior to sampling supports that these WNV infections were autochthonous. Studies on the long-term persistence of antibodies against WNV in horses are limited; however, detection for at least 15 months post-infection has been reported [22,73]. As an aside observation, transport of horses in 2020 may have been impacted by the lack of equestrian events during the COVID-19 pandemic. This was supported by only 20.7% of all horses, and 29.6% of WNV-positive horses, being transported further than 20 km from the holding in the previous year.

Within the study area, WNV seroprevalence differed significantly between counties and was higher than statistically expected in the more northern counties of Elbe-Elster (14%), Anhalt-Bitterfeld (8.4%), and Wittenberg (4.7%), whereas it was lower than statistically expected in the more southern counties of Central Saxony (3.7%), Meissen (3%), and Dresden (0%). This difference may be partly explained by the geographical structure of the northern counties in the North German Plain. Regarding the elevation profile, Elbe-Elster, Anhalt-Bitterfeld and Wittenberg are located between zero and 75 m above sea level (Elbtalniederung, Elbe-Mulde-Tiefland, and Fläming), while Central Saxony, Meissen and Dresden are predominantly located up to 450 m above sea level (Erzgebirge, Erzgebirgsvorland and Sächsisches Hügelland). On the one hand, the floodplains of the northern counties provide optimal environmental conditions for migratory birds, which are considered virus carriers after infection in their winter quarters in Africa or southern Europe [74]. Moreover, they provide favorable breeding sites for competent vectors. Due to the specific ecological interactions between bird and vector populations, bird protection areas have been highlighted as a potential risk factor for infection [75,76,77,78]. As the entire study area here was located in the same temperate climatic zone, the observed differences likely cannot be explained by the prevailing climatic conditions, of which temperature and precipitation have been identified as the most critical factors influencing the spread of WNV [77,79,80]. However, variations in temperature-dependent extrinsic incubation times (EIP) have been estimated within the study area [54,80]. The EIP describes the period needed for a biting mosquito to transmit the virus after its uptake during a preceding blood meal and, therefore, estimates the risk of virus transmission under local temperature conditions. An EIP of 10–15 days in the northern counties during the summer months may increase the risk of virus transmission compared to the EIP of up to 30 days in the southern counties [54].

The distribution of registered equine WNV infections in 2020 and 2021 [55] strengthens the suspicion that the virus is spreading increasingly in the damp meadow areas of the North German Plain. While eight WNV infections in Saxony and six infections in Brandenburg were registered in 2020, 14 infections in Brandenburg but only two infections in Saxony were reported in 2021 [81]. However, it must be considered that the number and distribution of reported WNV infections are subject to various factors. For instance, the vaccination of horses against WNV has been financially supported by the semi-governmental livestock insurance fund in Saxony since 2019 and for 2020, applications for reimbursement have been submitted for approximately 10% of all registered horses [82]. Since the effectiveness of vaccination in protecting against clinical disease has been repeatedly demonstrated [30,83,84], a higher vaccination coverage in the population may explain the lower number of reported cases.

The high seroprevalence (11.1%) in the city of Leipzig compared to the neighboring counties of Northern Saxony (4.4%) and Leipzig (3.5%) may be explained by an increased risk of virus transmission in the urban environment. Studies in the USA and Canada have associated urban landforms and demographic factors such as population density and urbanization with an increased risk of WNV infection in humans [85,86]. Conversely, no clear positive association between urbanization and WNV infections has been identified in Europe. Instead, there are reports of a positive association between WNV circulation and the presence of natural areas such as populated forests [87], wetlands [88], and river basins [89]. It must also be noted that only 18 horses in five holdings in Leipzig city were enrolled in the study, and further data are necessary to confirm or refute these results.

Concerning the three counties with no reported equine WNV infections in 2018/2019, no WNV infections in horses were registered in the transmission seasons of 2020/21 in Dresden (WNV seroprevalence 0%) and Central Saxony (WNV seroprevalence 3.7%). In Meissen (WNV seroprevalence 3.0%), clinical WNV infections were reported in two horses in September 2020. Thus, we demonstrated WNV seropositivity in counties without reported equine infections, showing that one cannot infer infection pressure from the number of registered WNV infections alone.

All serum samples were initially tested by a cELISA, which allows the species-independent detection of antibodies against the envelope protein of viruses belonging to the JEV serocomplex and TBEV. ELISAs are the preferred screening tools in live animals due to their speed, high throughput, high sensitivity, and ease of use [90]. WNV-specific antibodies can be detected in the blood of an infected horse as early as six to eight days post-infection [13]. While IgM antibodies presumably drop below the level of detection after about three months [73], IgG antibodies circulate for at least 15 months before they decline [15,22]. Thus, detection of IgM antibodies in six horses indicated recent infections. None of the horses had traveled outside of Germany, and five of six had not been transported more than 20 km from the holding in the past year, further supporting the theory that those infections occurred in the counties where they were registered (ABI, WB CS).

Six cELISA-positive or equivocal samples did not show neutralizing antibodies in the VNT. Theoretically, these results could be explained by the presence of antibodies to the PrM and E proteins of one of the three viruses (WNV, TBEV, USUV) at an early stage of infection. They compete for the binding site of the detection antibody in the kit but are non-neutralizing and therefore are not detected in the VNT. Another potential explanation could be the existing cross-reactivity with other flaviviruses unknown or not investigated in this study (e.g., Bagaza virus, Louping ill virus, JEV). As at present, the circulation of these viruses appears to be limited to southern Europe, the UK, and Norway [91,92,93], exposure within Germany seems unlikely. A final explanation for ELISA-reactivity in these samples is currently lacking.

Three other sera showed neutralizing antibodies against more than one of the tested flaviviruses without a titer difference of at least four log steps. This may indicate co-infection or sequential infection with different flaviviruses. Similar to previous descriptions in horses [50] and birds [94], respectively, two sera neutralized WNV and TBEV, while neutralizing antibodies against WNV and USUV were detected in one additional sample.

Thirty-five sera contained neutralizing antibodies against TBEV, with ND_50_ titers ranging from 40 to 2560. TBEV is transmitted by *Ixodes* ticks and is endemic throughout many countries of Central, Eastern and Northern Europe, as well as large parts of Northern and Central Asia [95,96]. In addition to human cases, TBEV infections have been detected in dogs [97], monkeys [98], and horses in Europe [99,100,101]. Based on the mandatory reporting of TBEV infections in humans, risk areas (described as regions with at least one human case per 100,000 inhabitants in five years) have been defined in Germany. These risk areas are mainly located in southern Germany (Bavaria, Baden Wurttemberg, southern Thuringia, and southern Hesse) but show a slow extension to the northeast (southern Saxony) and scattered areas in the northwest [102,103]. In horses, few epidemiological studies have been conducted primarily in the endemic areas [104,105]. Information on the spread of antibodies against TBEV in horses outside these areas is limited. In the present study, 12 of the 21 holdings with at least one TBEV-positive horse were located in known Saxonian risk areas, namely in Dresden, Meissen, and Central- Saxony. Six herds with single positive horses were located in districts not currently defined as risk areas, namely Leipzig district and Northern Saxony in Saxony, and Wittenberg and Anhalt-Bitterfeld in Saxony-Anhalt. In the Elbe-Elster district in Brandenburg, ten seropositive horses were detected in three holdings within a 5 km radius, indicating high TBEV activity in this county. While the counties immediately bordering Elbe Elster to the east (Oberspreewald-Lausitz, Oder-Spree, Spree-Neiße) have been designated as risk areas by the RKI for the first time in 2022, Elbe-Elster itself has not yet been defined as such.

Neutralizing antibodies against USUV were detected in eight samples from seven holdings. Those horses were born in Germany, did not travel abroad, and did not show clinical signs of neurological disease in the previous two years. WNV and USUV are antigenically closely related and have a similar transmission cycle, with *Culex* species as vectors, birds as amplification hosts, and horses and humans as dead-end hosts [106]. In addition, they often spread in the same environment [107,108]. Equine antibodies neutralizing against USUV were detected in surveillance studies in several European and African countries, where they occurred with or without concurrent neutralizing antibodies against WNV [47,52,109,110,111,112]. In Germany, USUV emerged for the first time in 2011 [113] and caused a massive die-off in Eurasian blackbirds in the Upper Rhine Valley in subsequent years. Aside from recognized epidemic areas, spanning southwestern Germany to the Dutch border, a higher occurrence of USUV-positive birds was found around Leipzig and Halle [114,115]. In horses, neutralizing antibodies against USUV were detected for the first time in one horse in the Berlin area in 2019 [61]. The detection of eight additional seropositive horses from six Eastern German counties supports the circulation of USUV in this region.

The second aim of the study was the identification of potential risk factors for WNV infection in Eastern Germany, and we detected six variables that were significantly associated with WNV seropositivity. At the animal level, ponies were found to be at lower risk of infection than Warmbloods. In previous studies, breed as a risk factor has been primarily attributed to differences in housing type, management, or geographic location of housing [76,116]. The correlation between breed and county of origin in the present study indicates that more ponies than statistically expected originated from counties with lower seroprevalences, which may explain the reduced risk of infection for them. An additional explanation for the lower risk of infection in ponies might be a biting preference of certain mosquitoes for different hair densities or sweat compositions [79]. Age was not identified as a risk factor in the present study, which may be explained by the recent initial introduction of the virus into the German horse population only two years before the study. In other studies, increasing age has been associated with increased risk of WNV seropositivity [110,116,117] and onset of clinical signs [26,118].

Horses kept in counties where equine WNV infections had been registered in previous years showed a higher risk of seropositivity. These results are consistent with previous studies in Italy and France [75], which showed higher seroprevalences in the immediate vicinity of recurrent WNV outbreaks and identified the occurrence of WN fever outbreaks in the previous year as a risk factor [78].

In the present study, horses with 24 h access to turn-out showed a higher risk of seropositivity than horses without permanent outdoor access, which is likely explained by a higher exposure to infectious bites. Similar results were shown in Tunisia [110], where equids kept outside had a significantly increased risk of being seropositive compared with those kept inside a stable. Interestingly, the presence of a shelter in the turn-out resulted in a higher risk of infection for horses in the present study when compared to horses without a shelter. This increased risk may partially be explained by the increased risk of permanent outdoor housing, as there was a moderate positive correlation between those variables.

Immune protection [119,120] and, thus, prevention of severe clinical disease in vaccinated as opposed to unvaccinated horses has been confirmed in several studies [30,83,84]. The observation in the present study of a reduced risk of seropositivity in non-vaccinated horses on holdings with a higher vaccination coverage is more difficult to explain. The moderate positive correlation between the number of horses kept on the holding, and the vaccination density might reflect a low spatial density of infectious vectors, as the risk of the individual horse being bitten decreases with the herd size.

Among mosquito control measures, only the use of fly sheets showed a significant effect in the regression model. Using a fly sheet in horses increased the risk of seropositivity compared to horses where a fly sheet was not used. At the same time, fly sheets were statistically more likely to be used on horses whose owners reported massive numbers or a lot of mosquitoes on the holding. We, therefore, assume that not the use of a flysheet but the high numbers of potentially infectious vectors, although only subjectively estimated, increased the risk of seropositivity. In addition, the results have to be interpreted with caution as fly sheets were only used in 10% of the horses. Mosquitoes of the *Culex pipiens* complex, which are the most potent WNV vectors are abundant in Germany [121]. Their susceptibility to the WNV [122,123] and the ability of *Culex spp*. to hibernate in subterranean habitats in Germany have already been described [124]. In 2019, WNV was detected for the first time in seven investigated mosquito pools obtained in the direct vicinity of registered equine WNV infections in Berlin [125]. A recent study from Saxony-Anhalt suggests that the virus might be able to overwinter within mosquitoes of the *Culex* spp. in Germany [126].

Limitations of our study mainly pertain to the lack of randomized sampling. A convenience sample was obtained, and we only invited horse owners with at least five registered equids to participate in the study. As only horses that were not vaccinated against WNV were included in the study, some horse owners with vaccinated as well as unvaccinated horses may have chosen not to participate. The geographically even distribution of sampled holdings within the study area probably compensates well for this limitation. A second possible limitation is the use of a questionnaire to obtain information for the risk factor analysis. Although the same veterinarian conducted all interviews on site at the time of sampling, and passports of all participating horses were examined, the accuracy of all statements cannot be fully verified. Therefore, possible individual perceptions of the questions and the accuracy of respondents’ statements can be seen as a limitation. In the context of logistic regression, independence between individual variables cannot be guaranteed. Influencing variables caused by the location apply to all horses of this holding and are therefore not independent of each other [127].

## 5. Conclusions

In conclusion, our results support the widespread geographical distribution of WNV in Eastern Germany in 2020 and the establishment of WNV infections in the local horse population. At the time of study, the level of seroprevalence differed among counties, but seropositive horses were also detected in counties without previously reported cases. Consequently, the study supports current vaccination recommendations [40]. In addition, our results also indicate circulation of TBEV and USUV in the study area. Concerning risk factors for WNV infections, we conclude that residence in counties with previously registered WNV infections and permanent outdoor housing pose an increased risk for WNV infections in unvaccinated horses. Ponies may have a reduced risk of infection; however, this finding requires additional study.

## Figures and Tables

**Table 1:** Study population and seroprevalence of WNV infection in Eastern Germany at horse level (**top**) and holding level (**bottom**). Of 940 horses included in the study, three horses with inconclusive serological results were excluded for the purpose of analysis.

**Federal State**	**County**	**Registered Horses** **(*n*)**	**Eligible** **Horses ^1^** ***n* (%)**	**Included** **Horses** ***n* (%)**	**WNV Positive** **Horses** ***n* (%)**
Saxony-Anhalt		4182	2151 (51.4)	237 (5.6)	15 (6.3)
	ABI	2412	1307 (54.2)	107 (4.4)	9 (8.4)
	WB	1770	844 (47.7)	130 (7.2)	6 (4.7) ^3^
Brandenburg	EE	2289	1301 (56.8)	136 (5.9)	19 (14.0)
Saxony		15,411	6738 (43.7)	567 (3.7)	20 (3.5)
	NS	3223	1385 (43.0)	137 (4.2)	6 (4.4) ^4^
	CS ^2^	4441	1967 (44.3)	164 (3.7)	6 (3.7)
	L	737	311 (42.2)	18 (2.4)	2 (11.1)
	LD	3378	1375 (40.7)	113 (3.4)	4 (3.5)
	MS ^2^	2672	1237 (46.3)	66 (2.5)	2 (3.0)
	D ^2^	960	463 (48.2)	69 (7.2)	0
Total		21,882	10,190 (46.6)	940 (4.3)	54 (5.8) ^5^
**Federal State**	**County**	**Registered Holdings** **(*n*)**	**Eligible** **Holdings ^6^** ***n* (%)**	**Included** **Holdings** ***n* (%)**	**Holdings with ≥1** **WNV-Positive** **Horses** ***n* (%)**
Saxony-Anhalt		1177	187 (15.9)	36 (3.1)	10 (27.8)
	ABI	649	114 (17.6)	16 (2.5)	5 (31.3)
	WB	528	73 (13.8)	20 (3.8)	5 (25.0)
Brandenburg	EE	634	108 (17.0)	13 (2.1)	5 (38.5)
Saxony		5902	633 (10.7)	78 (1.3)	12 (15.4)
	NS	1232	128 (10.4)	13 (1.1)	3 (23.1)
	CS ^2^	1630	200 (12.3)	27 (1.7)	2 (7.4)
	L	318	22 (6.9)	5 (1.6)	2 (40.0)
	LD	1311	132 (10.1)	14 (1.1)	3 (21.4)
	MS ^2^	1028	107 (10.4)	10 (1.0)	2 (20.0)
	D ^1^	383	44 (11.5)	9 (2.4)	0
Total		7713	928 (12.0)	127 (1.7)	27 (21.3)

^1^ Horses kept in holdings with at least five horses, ^2^ Counties without registered equine WNV infections in 2018/2019, ^3^ Two samples were excluded from the analysis as VNT results were inconclusive; ^4^ One sample was excluded from the analysis as VNT results were inconclusive; ^5^ Percentage calculated based on *n* = 937 samples, ^6^ Holdings with at least five registered horses; ABI: Anhalt-Bitterfeld; WB: Wittenberg; EE: Elbe-Elster; NS: Northern Saxony; CS: Central Saxony, L: Leipzig city, LD: Leipzig district, MS: Meissen; D: Dresden city.

**Table 2:** Univariate analysis (chi-square) of potential risk factors for WNV seropositivity (based on VNT results) in 937 horses in Eastern Germany. Only significant variables (*p* < 0.05) are shown.

Variable	χ^2^	df	*p*
Breed type	12.9	4	0.012
County	27.4	8	0.001
County—category ^1^	7.7	1	0.005
No. of horses in the holding	59.8	33	0.003
Type of housing	7.2	1	0.007
Type of turn-out	8	2	0.018
No. of additional horses within the turn-out	67.6	17	<0.001
Presence of outdoor shelter	9.3	1	0.002
WNV-vaccination density	54.4	36	0.025
Use of self-made insect repellent	13.5	1	<0.001
Frequency of repellent use in turn-out	9.4	3	0.024
Use of fly sheets	18	1	<0.001
Type of fly sheet	46.2	3	<0.001
Weekly water change in troughs in turn-out	5.1	1	0.024

χ^2^: Chi^2^ value; df: degrees of freedom; *p*: significance; ^1^ With/without registered equine WNV infections in 2018/2019.

**Table 3:** Logistic Regression model for the association of potential risk factors with WNV seropositivity in 937 horses in Eastern Germany. Only variables with significant categories (*p* < 0.05) are shown.

Variable	Category	Exp (B)	*p*	95% CI	
				Lower	Upper
Breed type	Warmblood	Ref.			
	Draft horse	1.92	0.383	0.4	8.2
	Thoroughbred	2.27	0.216	0.6	8.3
	Pony	0.29	0.013	0.1	0.8
	Donkey	1.59	0.733	0.1	22.8
County	Without registered equine WNV infections in 2018/2019 ^1^	Ref.			
	With registered equine WNV infections in 2018/2019 ^2^	3.91	0.003	1.6	9.7
Type of housing	<24 h access to turn-out	Ref.			
	Permanent outdoor housing	2.63	0.033	1.1	6.4
Presence of outdoor shelter	No	Ref.			
	Yes	3.02	0.010	1.3	7.0
WNV-vaccination density	In percent	0.97	0.010	0.9	1.0
Use of fly sheets	No	Ref.			
	Yes	7.22	<0.001	2.7	19.0

^1^ Central Saxony; Meissen; Dresden city; ^2^ Anhalt-Bitterfeld; Wittenberg; Elbe-Elster; Northern Saxony; Leipzig city; Leipzig district; Exp(B): Odds ratio (the exponentiation of the B coefficient); CI: confidence interval; Ref.: reference variable.

## Data Availability

The study results’ data are included in the article or Supplementary Data. Further specific information regarding the dataset analyzed during the study can be obtained from the corresponding author on reasonable request.

## Supplementary Materials

The following are available online at: https://www.mdpi.com/article/10.3390/v14061191/s1, Table S1: Individual holdings included in the study and results of serological analysis (*n* = 937 horses); Table S2: Descriptive statistics for horses included in the study, and distribution of answers from a questionnaire concerning potential risk factors for WNV seropositivity (*n* = 937); Table S3: Results of virus neutralization assays (VNT) for 106 horses testing positive or equivocal by competitive ELISA; Table S4: Complete univariate analysis (chi-square) of potential risk factors for WNV seropositivity in 937 horses; Table S5: Logistic regression model (complete data) for the association of potential risk factors with WNV seropositivity in 937 horses; Figure S1: standardized questionnaire for epidemiological data collection.
