# Supplementary material for: Seroprevalence and Risk Factors for Equine West Nile Virus Infections in Eastern Germany, 2020
Source: Viruses. 2022 May 30;14(6):1191. doi: 10.3390/v14061191 (PMC9229339; doi:10.3390/v14061191)
Supplement: Supplementary file 1 [file viruses-14-01191-s001.zip › Suppl.data_proofreading/Questionnaire_final.pdf]

# Questionnaire: Seroprevalence of equine WNV infections in Eastern Germany, 2020

Dear horse owner,

For the assessment of laboratory results, we need some information about your horse and ask you to answer the following questions. In addition, to ensure direct communication and transmit the test results, we need your complete contact information. The information provided is crucial for the complete execution of the study and will be kept strictly confidential.

Please refer to the 'Informed consent Statement for Participation in a Scientific Study' for more information on data protection.

Please note that only horses that have not been vaccinated against the West Nile virus can participate in this study.

**Contact:**

Name: ___________________________________First Name: _______________________________

Address: ___________________________________________________________________________

Address of the holding: ______________________________________________________________

Phone: _____________________________________________________________________________

Mail: _______________________________________________________________________________

**Identification horse/s:** (name, date of birth, signalment, ID)

1: _________________________________________________________________________________

2: _________________________________________________________________________________

3: _________________________________________________________________________________

4: _________________________________________________________________________________

**Notes on answering:**

Please take a few minutes to fill out this questionnaire. There is no judgment and no right or wrong answer. Only truthful answers will allow us to analyze the data correctly. For each question, please choose the solution that best fits your horse. Please check only one possible answer. You may indicate alternatives to the suggested categories under 'other' for some questions. For other questions, there is the possibility to explain your answer under 'further information.' Please check one of the answers for those questions as well. If you fill in the questionnaire for more than one horse, please write the name of the horse or the ID number from the contact data behind the respective answer. If you have already indicated the complete signalment of the horses in the contact data, you can skip these questions in the information as of question 10 is identical for all horses named by you in the questionnaire, one check mark per question is sufficient.

**Details of your horse(s):**

1. **My horse is a:**

☐ Draft horse or draft horse cross

☐ Warmblood (incl. heavy warmbloods)

☐ Thoroughbred

☐ Pony

☐ Donkey/mule

☐ Other: ________________________________

2. **My horse is a:**

☐ Mare

☐ Stallion

☐ Gelding

3. **What coat color type does your horse correspond to?**

☐ Dark (black/dark bay/bay)

☐ Chestnut

☐ Light (roan/dun/palomino)

☐ Very light or white (cremello/white)

☐ Spotted (Tobiano/Overo/Appaloosa)

☐ Other: ___________________________________

4. **Was your horse bred/born in Germany?**

☐ Yes

☐ No

If not, where? ______________________________________________________________

5. **Did your horse travel outside of Germany in the past two years?**

☐ Yes

☐ No

If yes, where to? ____________________________ When? ______________________

6. **Did your horse show any clinical signs of neurologic disease in the past two years (e.g., ataxia, stiff gait, muscle fasciculations)?**

☐ Yes

☐ No

If yes, when? _________________________________________________________________

Which clinical signs? ___________________________________________________________

_____________________________________________________________________

**Holding and Primary use:**

7. **How many horses are kept in the holding where your horse is kept?**

   ______________________________________________________________________

8. **How is your horse housed during the mosquito season (April to November)?**

   ☐ Stabled without turn-out

   ☐ Stabled with a freely accessible turn-out

   ☐ Stabled with turn-out time <12h/day

   ☐ Stabled with turn-out time >12h/day

   ☐ Permanent outdoor housing

   Further information: ___________________________________________________

9. **What is your horse's turn-out like?**

   ☐ dry lot (fenced, usually close to the stable, dry lot without grass cover)

   ☐ pasture (fenced, mostly natural pasture with grass cover)

   ☐ combination of both (e.g., open stable with access to pasture)

   Further information: ___________________________________________________

10. **Are there additional horses in the turn-out?**

    ☐ Yes

    ☐ No

    If yes, how many? _____________________________________________________

11. **Is an outdoor shelter provided in the turn-out?**

    ☐ Yes

    ☐ No

    Type of shelter: ______________________________________________________

12. **Is there stagnant water within approximately 1 km of the holding?**

    ☐ Yes

    ☐ No

    Further information: __________________________________________________

13. **Has your horse been transported further than 20 km from the holding in the last 12 months?**

    ☐ Yes

    ☐ No

    If yes, where to? _____________________________________________________

**14. What is the primary use of your horse?**

- ☐ Leisure
- ☐ Sport (Dressage/Show jumping/Driving/________________________________)
- ☐ Breeding
- ☐ Retired
- ☐ Other: ______________________________________________________________

**15. Where is your horse primarily exercised?**

- ☐ Exclusively in the arena
- ☐ Mainly in the arena
- ☐ Mainly trail riding
- ☐ Exclusively trail riding
- ☐ No exercise

Further information: _________________________________________________________

**16. How many horses on the holding are vaccinated against West Nile virus?**

- ☐ None
- ☐ Some → How many? ____________________________________
- ☐ All of them

**17. To your knowledge, has WNV infection been diagnosed in a horse (on your or a neighboring holding) within the last 2 years?**

- ☐ Yes
- ☐ No

If yes, where and when? _________________________________________________________

_____________________________________________________________

**Mosquito control measures in the holding:**

18. **How would you estimate the number of mosquitoes in your horse´s holding?**

☐ Massive numbers

☐ A lot

☐ Few

☐ None

19. **Do you use an insect repellent on your horse?**

☐ Yes

☐ No → Please continue with question 22.

20. **What type of insect repellent do you use?**

☐ Homemade product, active ingredient: ______________________________

☐ Commercial product, brand name: ______________________________

21. **In which situation and how frequently do you use the insect repellent?**

| | **Allways (at least once daily)** | **Mostly (at least once weekly)** | **Rarely (Less than once a week)** | **Never** |
|---|---|---|---|---|
| **While stabled** | ☐ | ☐ | ☐ | ☐ |
| **While exercising** | ☐ | ☐ | ☐ | ☐ |
| **In the turn-out** | ☐ | ☐ | ☐ | ☐ |
| **During transport** | ☐ | ☐ | ☐ | ☐ |
| **Others (please specify):** | ☐ | ☐ | ☐ | ☐ |

22. **Do you use a fly sheet on your horse during mosquito season?**

☐ Yes

☐ No → Please continue with question 24.

23. **What type of fly sheet do you use?**

☐ regular fly sheet

☐ patterned fly sheet (e.g., zebra print)

☐ sweet itch sheet

☐ Other: ______________________

**24. Which of the following measures are carried out in your horse´s holding?**
(Multiple responses possible)

| | **Apply** | **Does not apply** |
|---|---|---|
| Use of automatic waterers | ☐ in the stable<br>☐ in the turn-out | ☐ in the stable<br>☐ in the turn-out |
| Use of buckets and/or troughs | ☐ in the stable<br>☐ in the turn-out | ☐ in the stable<br>☐ in the turn-out |
| complete change of drinking water in troughs (at least weekly) | ☐ in the stable<br>☐ in the turn-out | ☐ in the stable<br>☐ in the turn-out |
| PVC strips on door openings | ☐ | ☐ |
| Fly screens in windows | ☐ | ☐ |
| Horsefly traps | ☐ | ☐ |
| Electric insect traps | ☐ | ☐ |
| Sticky tape fly traps | ☐ | ☐ |
| Traps with attractant | ☐ | ☐ |
| Other (please specify) | | |

**Is there any other information about your horse you'd like to share that we haven't asked you yet? Here is room for your response:**

________________________________________________________________________

________________________________________________________________________

________________________________________________________________________

May we contact you for participation in further studies concerning West Nile virus infections in horses?

☐ Yes, please use the contact details above.

☐ No, I am not interested in any further contact.

You made it ...Thank you very much for your participation!
